# A Family Affected by a Life-Threatening Erythrocyte Defect Caused by Pyruvate Kinase Deficiency With Normal Iron Status: A Case Report

**DOI:** 10.3389/fgene.2020.560248

**Published:** 2020-10-28

**Authors:** Karolina Maciak, Anna Adamowicz-Salach, Jaroslaw Poznanski, Monika Gora, Jan Fronk, Beata Burzynska

**Affiliations:** ^1^Institute of Biochemistry and Biophysics, Polish Academy of Sciences, Warsaw, Poland; ^2^Department of Pediatrics, Hematology and Oncology, Medical University of Warsaw, Warsaw, Poland; ^3^Faculty of Biology, Institute of Biochemistry, University of Warsaw, Warsaw, Poland

**Keywords:** congenital non-spherocytic hemolytic anemia, pyruvate kinase deficiency, iron metabolism, hepcidin, *TMPRSS6* (matriptase-2)

## Abstract

**Background:**

Red cell pyruvate kinase deficiency (PKD) is a defect of glycolysis causing congenital non-spherocytic hemolytic anemia. PKD is transmitted as an autosomal recessive trait. The clinical features of PKD are highly variable, from mild to life-threatening anemia which can lead to death in the neonatal period. Most patients with PKD must receive regular transfusions in early childhood and as a consequence suffer from iron overloading.

**Patient:**

Here, we report a Polish family with life-threatening hemolytic anemia of unknown etiology. Whole exome sequencing identified two heterozygous mutations, c.1529 G > A (p.R510Q) and c.1495 T > C (p.S499P) in the *PKLR* gene. Molecular modeling showed that the both *PKLR* mutations are responsible for major disturbance of the protein structure and functioning. Despite frequent transfusions the patients do not show any signs of iron overload and hepcidin, a major regulator of iron uptake, is undetectable in their serum. The patients were homozygous for the rs855791 variant of the *TMPRSS6* gene which has earlier been shown to down-regulate iron absorption and accumulation.

**Conclusion:**

The lack of iron overload despite a reduced level of hepcidin in two transfusion-dependent PKD patients suggests the existence of a hepcidin-independent mechanism of iron regulation preventing iron overloading.

## Introduction

Pyruvate kinase deficiency (PKD) is a common cause of hereditary non-spherocytic hemolytic anemia. Pyruvate kinase is crucial in the energy metabolism of red blood cells (RBC) because it catalyzes one of the two steps of ATP production in glycolysis, the sole energy-producing pathway in erythrocytes. Its deficiency impairs glycolysis and leads to the accumulation of upstream metabolites. PKD is autosomal recessive and manifests with hemolysis of highly variable degree in homozygotes or in compound heterozygotes (Zanella et al., 2007). PKD diagnosis is based on measurements of the enzyme activity, although normal values are sometimes obtained due to incomplete leukocyte removal or contamination with donor cells in transfused patients (Grace et al., 2015). Most patients with PKD must receive regular transfusions in early childhood and as a consequence suffer from iron overloading (Zanella et al., 2007). Moreover, even in non-regularly transfused patients, iron overload has been reported (Van Beers et al., 2019).

Iron metabolism is regulated in a highly complicated manner, with the hormone hepcidin playing a major role (Wang and Babitt, 2019). One of the factors affecting hepcidin level is a serine protease, matripase-2 (TMPRSS6). Recently, a new form of hereditary anemia called iron refractory iron deficiency anemia (IRIDA), due to mutations in the *TMPRSS6* gene, has been described (Finberg, 2009). Genome-wide association studies have shown that SNP rs855791, which causes the V736A amino acid substitution in matripase-2, is associated with variations of serum iron, transferrin saturation, and hemoglobin and erythrocyte traits. The iron parameters are significantly higher in homozygotes 736A than in 736V (Nai et al., 2011). Some reports show that the 736A variant of matripase-2 inhibits hepcidin more efficiently than 736V (Nai et al., 2011; Valenti et al., 2012), while other studies found no correlation between the rs855791 polymorphism and hepcidin levels, suggesting that matripase-2 affects the iron parameters by a hepcidin-independent mechanism (Traglia et al., 2011; Galesloot et al., 2013).

In this paper, we report a Polish family with two sons carrying a compound defect of the *PKLR* gene causing a life-threatening hemolytic anemia. Despite frequent transfusions the boys have a normal iron level and no symptoms of iron poisoning. Both boys are homozygotic for the 736A variant of matripase-2, while none of the polymorphisms found in other genes related to iron metabolism have been reported to affect iron loading. Among the homozygous polymorphisms of known genes related to iron metabolism found in the both patients’ exomes only the 736A variant of matripase-2 has earlier been reported to affect iron loading.

## Case Presentation

Two brothers, 12 and 8 years old, have been under the care of the outpatient department of a hematology clinic since birth due to hemolytic anemia of unknown etiology. The older brother has been transfused 67 times due to prominent anemia with jaundice. At the age of 9, he underwent partial splenectomy, after which the frequency of transfusions could be reduced, but only transiently. The second brother presents similar clinical features of severe hemolytic anemia. He has been hospitalized 21 times for packed RBC transfusions. A third brother died shortly after birth because of massive hemolytic anemia. The hematological parameters of the patients are shown in Table 1. The RBC parameters do not unequivocally indicate the cause of the anemia. Unexpectedly, despite the frequent transfusions, iron, ferritin, TIBC and TSAT levels are within norm, while serum hepcidin is below the limit of detection. There is no family history of blood diseases.

**TABLE 1:** Hematological parameters of the probands.

**Case**	**Age**	**WBC[10^9^/L]**	**RBC [10^12^/L]**	**Ret [%]**	**Ret [10^9^/L]**	**Ht [%]**	**Hb [g/L]**	**MCV [10^–15^L]**	**RDW [%]**	**Ferritin [10^–6^g/L]**	**Iron [10^–5^g/L]**	**TIBC [10^–5^g/L]**	**TSAT [%]**	**Pyruvate kinase [U/gHb]**	**Hepcidin-25 [nM]**
Normal value	–	4.0–12.0	4.2–5.4	0.6–2.6	42–70	37–47	120–160	81–99	11–15	5.6–216	20–145	173–356	20–50	9.1–23.7	2.1*
Brother 1	12 years	6.2	2.6	24.2	639.9	24.7	76	95.7	23.6	28.2	73	389	18.8	7.5	<0.5
Brother 2	9 years	7.3	3.1	9.7	290.7	27.8	93	88.5	17.1	36.8	95	374	25.4	6.1	<0.5
Brother 3	18 h	51.0	2.3	nd	nd	26.5	53	nd	nd	nd	nd	nd	nd	nd	nd

WBC, white blood cells; RBC, red blood cells; Ret, Reticulocytes; Ht, hematocrit; Hb, hemoglobin; MCV, mean corpuscular volume; RDW, red blood cell distribution width; TIBC, total iron binding capacity; TSAT, transferrin saturation; nd, not determined. *Median; range: p 2.5–p 97.5 = < 0.5–13.0 nM (http://www.hepcidinanalysis.com).

## Materials and Methods

### Patients

Clinical evaluation of the patients were performed at the Department of Pediatrics, Hematology and Oncology, Medical University of Warsaw.

### Methods

DNA was prepared from EDTA blood samples using the QIAamp DNA Mini Kit (Qiagen, Hilden, Germany). Whole exome sequencing (WES) was performed on the two affected brothers (CeGaT GmbH, Tübingen, Germany). Exome libraries were prepared using SureSelectXT Library Prep Kit (Agilent, Santa Clara, CA). Enrichment was performed using SureSelect Human All Exon V7 Kit (Agilent). Sequencing was performed on an Illumina sequencer (NovaSeq 6000) with 2 × 100 bp sequencing mode and 12 Gb output per sample. Demultiplexing of the sequencing reads was performed with Illumina bcl2fastq (version 2.19). Adapters were trimmed with Skewer (version 0.2.2) (Jiang et al., 2014). Trimmed raw reads were aligned to the human reference genome (hg19-cegat) using the Burrows-Wheeler Aligner (version 0.7.17-cegat) (Li and Durbin, 2009). ABRA (version 2.18) (Mose et al., 2014) was used for local realignment of reads in target regions to enhance performance for indel detection. In the reference hg19-cegat the pseudoautosomal regions on chromosome Y were masked (chrY:10001-2649520,chrY:59034050-59363566). Reads that could be aligned to more than one locus with the same mapping score were discarded. Duplicated reads, which most likely originated from the same PCR amplicon, were discarded using SAMtools (version 0.1.18) (Li et al., 2009). A proprietary software was used for variant detection. The reads were annotated and analyzed using Ensembl v92, RefSeq Curated (20180710), CCDS r22, dbSNP151, and GnomAD 2.1 databases. The lists also include variants with low frequencies (observed frequency of the alternative allele down to 2% of sequenced reads).

Two mutations detected in the *PKLR* gene (NM_000298.6) were verified using Sanger sequencing in the probands and also were assessed in both parents. Bioinformatic analysis using MutationTaster (Schwarz et al., 2014), PROVEAN (Choi et al., 2012), PolyPhen-2 (Adzhubei et al., 2010), and M-CAP (Jagadeesh et al., 2016) predicted deleterious effects of both these mutations. Both mutations were also classified according to the American College of Medical Genetics and Genomics (ACMG) guidelines (Richards et al., 2015; Table 2). Serum hepcidin was determined (Hepcidinanalysis.com, Laboratory Medicine, Nijmegen, Netherlands) by weak cation exchange chromatography followed by time-of-flight mass spectrometry (WCX-TOF MS).

**TABLE 2:** Prediction of pathogenicity of PKLR mutations.

**Mutation**	**Type**	**Amino acid substitution**	**Prediction**				**ClinVar classification**	Variant assessment*
			**MutationTaster**	**PROVEAN**	**PolyPhen2**	**M-CAP**		
c.1495 T > C	Missense	S499P	“Disease causing”	−2.464	0.973	0.193	nd	Pathogenic
c.1529 G > A	Missense	R510Q	“Disease causing”	−3.723	1.00	0.177	Pathogenic	Pathogenic

MutationTaster is a web-based application for evaluation of DNA sequence variants for their disease-causing potential (http://www.mutationtaster.org/). PROVEAN predicts whether an amino acid substitution or indel has an impact on the biological function of a protein; scores equal to or below −2.5 are considered “deleterious” (http://provean.jcvi.org). PolyPhen2 predicts whether an amino acid substitution or indel has an impact on the biological function of a protein; scores range from 0 (tolerated) to 1 (deleterious) (http://genetics.bwh.harvard.edu/pph2/). M-CAP is a pathogenicity classifier for rare missense variants in the human genome; scores above 0.025 are considered “deleterious” (http://bejerano.stanford.edu/mcap/). nd, no data. *Pathogenicity was assessed following the guidelines of the American College of Medical Genetics and Genomics and Association for Molecular Pathology.

To characterize the likely effects of the two *PKLR* mutations, molecular modeling was performed. A maximal-length structure of human pyruvate kinase was modeled by combining eight structures available in PDB (4ima, 4ip7, 2VGB, 6B6U, 2G50, 3GR4, 3GQY, and 3U2Z) using Yasara Structure Package (Krieger et al., 2002). The final model representing homotertrameric structure covered amino acid residues 36–574. The effect of amino acid replacements on the protein stability was assessed with FoldX (Schymkowitz et al., 2005).

## Results

To facilitate full diagnosis, WES was performed on the two brothers. The WES data showed 42,823 (brother 1) and 41,937 (brother 2) SNPs along with 5402 (brother 1) and 5,137 (brother 2) small insertions or deletions. They were inspected for mutations in the 71 known hereditary anemia genes (Russo et al., 2018). In both brothers we found two heterozygous mutations, c.1529 G > A (p.R510Q) and c.1495 T > C (p.S499P), in the *PKLR* gene encoding the pyruvate kinase isoenzyme expressed in erythrocytes. The c.1529 G > A mutation is the most common *PKLR* mutation in the northern and central European population^1^ with a frequency of 0.0007 (rs113403872). The c.1495 T > C mutation is not included in databases such as 1,000 Genomes, dbSNP, ExAC, gnomAD, or HGMD. Very recently, this mutation was mentioned for the first time by Bianchi et al. (2020). These mutations were confirmed by Sanger sequencing (Supplementary Figure 1), and they were also found individually in the heterozygous state in the asymptomatic mother (p.R510Q) and father (p.S499P). Bioinformatic analysis using MutationTaster, PROVEAN, PolyPhen-2 and M-CAP predicted deleterious effects of both these mutations (Table 2).

**FIGURE 1:**
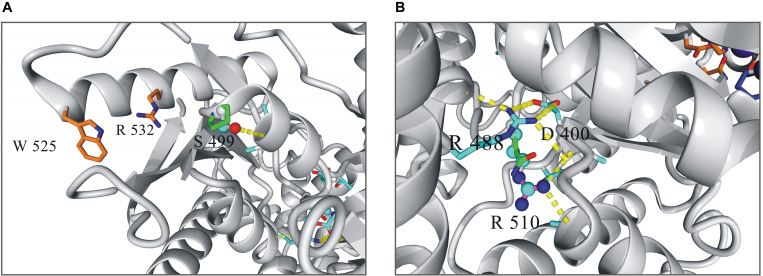
Local structure of human pyruvate kinase in the vicinity of residues 499 **(A)** and 510 **(B).** Wild-type residues in the mutated positions are shown as balls-and-sticks model, mutated variants as sticks.

The both substitutions, S499P and R510Q, were found to affect markedly the stability and functioning of the enzyme (Figure 1). The hydroxyl group of S499, which is located at the N-terminus of helix 23 (S499-V506), forms a hydrogen bond with the main chain amide group of A502. This interaction represents the common N-capping mechanism stabilizing the first turn of the helix. The S499P replacement destabilizes the protein structure by 2.6 kcal/mol, mainly due to the loss of the hydrogen bond and to van der Waals clashes with proximal residues. S499P should additionally interfere with allosteric activation, in which the proximal W525 and R532 are involved. R510, in turn, is involved in a network of interactions stabilizing the core structure of PKLR. It forms two hydrogen bonds positioning the short loop that separates helix 41 and sheet 2. These interactions are unique to arginine, and any replacement of R510 decreases the protein stability. The R510Q replacement destabilizes PKLR by 3 kcal/mol. It should be noted that the resulting local destabilization of the protein may propagate to proximal metal-binding sites, thus affecting the catalytic activity of PKLR.

Because of the serious defects of PKLR, our patients present with an extremely severe form of anemia requiring regular transfusions. Nevertheless, they do not show elevated serum iron parameters nor any signs of iron overload. To solve this conundrum the WES results were inspected for possible variants that could affect iron metabolism. Indeed, the both brothers carried the rs855791 (V736A) polymorphism in the *TMPRSS6* gene (NM_001289001.1) encoding matripase-2 in homozygous state (Supplementary Figure 2). This particular polymorphism in the *TMPRSS6* gene has earlier been found to affect iron parameters through an as yet uncertain mechanism (Nai et al., 2011; Traglia et al., 2011; Valenti et al., 2012; Galesloot et al., 2013). A genome-wide association study has indicated that the rs855791 polymorphism modulates erythropoiesis and iron metabolism (Benyamin et al., 2009). It has also been reported that the 736A variant of TMPRSS6 correlates with low hepcidin level (Nai et al., 2011), indicating the likely mechanism of the rs855791 influence on iron status, albeit other studies found no such correlation, which suggested a hepcidin-independent mode of matripase-2 action (Traglia et al., 2011; Galesloot et al., 2013).

An alternative explanation would be that an iron-status regulator other than matripase-2 was responsible. Among all the genes known to be related to iron metabolism we found, in addition to the rs855791 SNP, 112 other cases of single nucleotide variants (SNV) and indels present in the both probands in the homozygous state (Supplementary Table 1). Unfortunately, none of these variants have so far been found to affect iron metabolism, therefore their relevance to the present case can only be speculative.

## Discussion and Conclusion

Iron refractory iron deficiency anemia (IRIDA) is a hereditary recessive anemia due to a defect in the *TMPRSS6* gene encoding a type II transmembrane serine protease matriptase-2 (Wang and Babitt, 2019). Matripase-2 negatively regulates the production of hepcidin, the main iron regulatory hormone, and some *TMPRSS6* mutations are associated with a significant decrease in the concentrations of iron and hemoglobin, and the mean corpuscular volume of RBC. Moreover, in the European cohort of microcytic iron deficiency anemia (De Falco et al., 2013), some patients have no *TMPRSS6* mutations and also show very low or undetectable hepcidin level, which suggests yet another pathophysiological mechanism.

The most common glycolytic enzymopathy and an important cause of hereditary, non-spherocytic hemolytic anemia is a defective erythrocytic pyruvate kinase (PK). PK-deficient patients usually develop iron overload due to a chronic hemolysis, ineffective erythropoiesis and transfusion therapy. In both our patients serum hepcidin was extremely low, in accordance with the reported correlation with the 736A matripase-2 variant (Nai et al., 2011), but contrary to expectations in view of the normal iron parameters of the patients. It therefore seemed likely that the postulated but as yet undefined hepcidin-independent mechanism of matripase-2 influence on iron metabolism was critical in preventing the negative side-effects of massive transfusions in the two patients. The participation of an iron-status regulator other than matripase-2 could offer an alternative explanation, but none of the polymorphisms in other iron-metabolism-related genes found in our patients could be assigned such a function basing on the available data.

Currently, in PKD and other hereditary hemolytic anemias the treatment is mostly supportive and includes packed RBC transfusions, which frequently results in iron overload. Moreover, anemic patients with PKD develop an iron overload even in the absence of chronic blood transfusions (Van Beers et al., 2019). Also those affected by thalassemia intermedia, who do not require transfusions, show the symptoms of iron overload (Rivella, 2019). This is due to anemic stress which provokes ineffective erythropoiesis causing increased absorption and tissue accumulation of iron. Ineffective erythropoiesis and low or inappropriately normal hepcidin levels are noted in so called iron-loading anemias (Pagani et al., 2019). Indeed, low levels of hepcidin have been found in PK-deficient patients reflecting the effect of ineffective erythropoiesis on hepcidin production (Mojzikova et al., 2014). In our PKD patients with the matripase-2 736A variant we do observe an elevated level of reticulocytes and low level of hepcidin, yet despite that there are no symptoms of iron overload. Thus, these results do not support a role for hepcidin in the prevention of iron overloading in our patients.

The NGS approach offers insights into the etiopathophysiology of the disease and can facilitate accurate diagnosis enabling better care of the patient (Di Resta and Ferrari, 2018). Since the clinical presentation of different types of congenital hemolytic anemia may be similar, molecular characterization can in some cases be essential for proper diagnosis.

In summary, we show that severe PKD is caused by a compound effect of the S499P and R510Q mutations in PKLR. We also present the very first case, as far as we know, of severe PK-deficient patients without an accompanying transfusional iron overload. This lack of iron over-accumulation is possibly due to a beneficial effect of the rs855791 *TMPRSS6* polymorphism by serum hepcidin independent mechanism.

## Data Availability Statement

All datasets generated for this study are included in the article/Supplementary Material, further inquiries can be directed to the corresponding authors.

## Ethics Statement

The studies involving human participants were reviewed and approved by the Ethics Committee of the Medical University of Warsaw. Written informed consent to participate in this study was provided by the participants’ legal guardian/next of kin. Written informed consent was obtained from the minor(s)’ legal guardian/next of kin for the publication of any potentially identifiable images or data included in this article.

## Author Contributions

KM, MG, and BB carried out experiments and wrote the manuscript. AA-S recruited the patients and was responsible for their clinical care. JP performed protein structure modeling. BB and JF interpreted data and edited the final version of the manuscript. BB supervised the study. All authors contributed to the article and approved the submitted version.

## Conflict of Interest

The authors declare that the research was conducted in the absence of any commercial or financial relationships that could be construed as a potential conflict of interest.

## Footnotes

1 https://databases.lovd.nl/shared/genes/PKLR
