# Supplementary material for: A Family Affected by a Life-Threatening Erythrocyte Defect Caused by Pyruvate Kinase Deficiency With Normal Iron Status: A Case Report
Source: Front Genet. 2020 Oct 28;11:560248. doi: 10.3389/fgene.2020.560248 (PMC7655982; doi:10.3389/fgene.2020.560248)
Supplement: Supplementary file 1 [file Data_Sheet_1.docx]

**Supplementary Material**

**Title:** A family affected by a life-threatening erythrocyte defect caused by pyruvate kinase deficiency with normal iron status: A case report

**Authors:** Karolina Maciak, Anna Adamowicz-Salach, Jaroslaw Poznanski, Monika Gora, Jan Fronk and Beata Burzynska

# Supplementary Figures and Tables

# Supplementary Figures


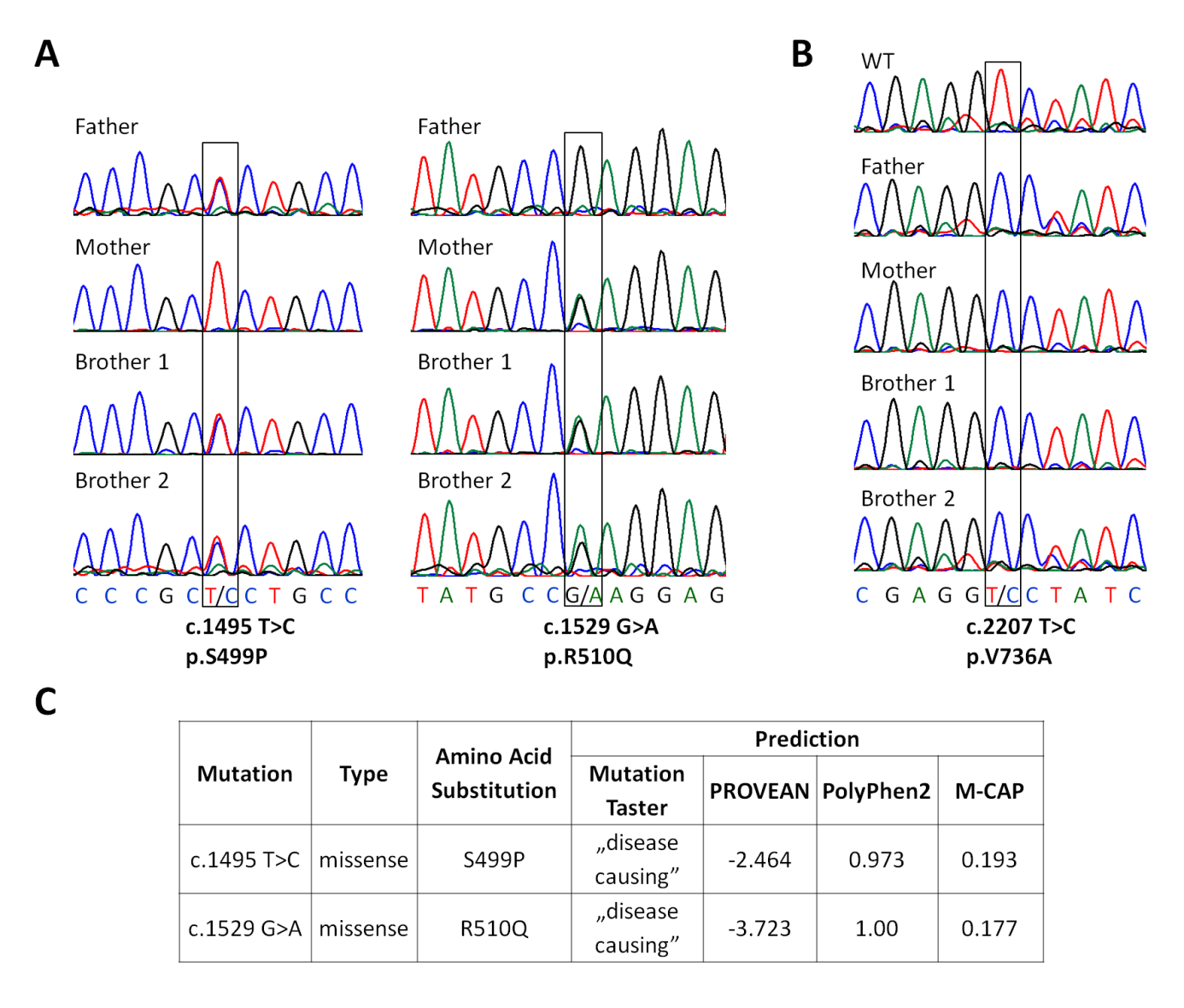


**Supplementary Figure 1.** Nucleotide sequence of fragments of the *PKLR* gene in the family studied.


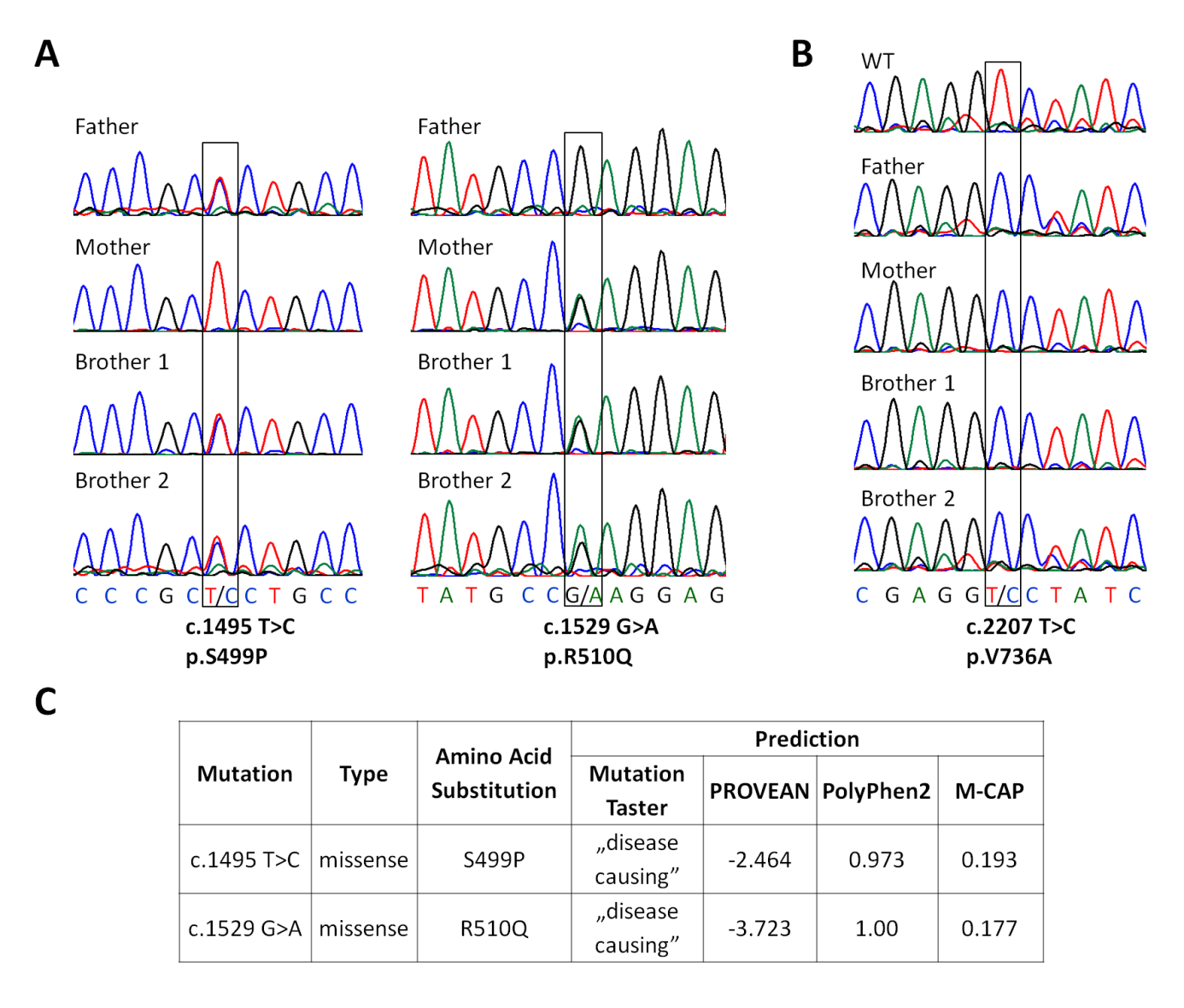


**Supplementary Figure 2.** Nucleotide sequence of fragments of the *TMPRSS6* gene in the family studied

- 1. **Supplementary Table**

**Supplementary Table 1.** Homozygotic variants in iron metabolism genes detected in two brothers.

| Gene | Transcript refseq ID | Protein | Base change | Consequence | aa change | dbSNP |
| --- | --- | --- | --- | --- | --- | --- |
| *ABCB6* | NM_005689.3 | ATP-binding cassette sub-family B member 6, mitochondrial | c.117G>A | synonymous | p.= | rs1109866 |
| *ABCB7* | NM_001271699.3 | ATP-binding cassette sub-family B member 7, mitochondrial | ND |  |  |  |
| *ABCG2* | NM_001257386.2 | Broad substrate specificity ATP-binding cassette transporter ABCG2 | c.1648-31T>C | intronic |  | rs2231162 |
|  |  |  | c.1367+20G>A | intronic |  | rs2231153 |
| *ACO1* | NM_001278352.1 | Cytoplasmic aconitate hydratase | ND |  |  |  |
| *ACO2* | NM_001098.2 | Aconitate hydratase, mitochondrial | c.670C>T | synonymous | p.= | rs1799932 |
| *ALAD* | NM_000031.5 | Delta-aminolevulinic acid dehydratase | ND |  |  |  |
| *ALAS1* | NM_000688.5 | 5-aminolevulinate synthase, nonspecific, mitochondrial | ND |  |  |  |
| *ALAS2* | NM_001037967.4 | 5-aminolevulinate synthase, erythroid-specific, mitochondrial | ND |  |  |  |
| *ATP13A2* | NM_001141974.2 | Cation-transporting ATPase 13A2 | ND |  |  |  |
| *ATP6AP1* | NM_001183.5 | V-type proton ATPase subunit S1 | ND |  |  |  |
| *ATP6V0B* | NM_001294333.1 | V-type proton ATPase 21 kDa proteolipid subunit | c.259+25A>C | intronic |  | rs12410334 |
| *ATP6V0C* | NM_001198569.2 | V-type proton ATPase 16 kDa proteolipid subunit | ND |  |  |  |
| *ATP6V0A1* | NM_001130021.3 | V-type proton ATPase 116 kDa subunit a isoform 1 | ND |  |  |  |
| *ATP6V0A2* | NM_012463.3 | V-type proton ATPase 116 kDa subunit a isoform 2 | ND |  |  |  |
| *ATP6V0A4* | NM_020632.2 | V-type proton ATPase 116 kDa subunit a isoform 4 | c.5T>C | missense | p.V2A | rs10258719 |
|  |  |  | c.1812T>C | synonymous | p.= | rs3807154 |
| *ATP6V0D1* | NM_004691.4 | V-type proton ATPase subunit d 1 | ND |  |  |  |
| *ATP6V0D2* | NM_152565.1 | V-type proton ATPase subunit d 2 | ND |  |  |  |
| *ATP6V0E1* | NM_003945.3 | V-type proton ATPase subunit e 1 | ND |  |  |  |
| *ATP6V0E2* | NM_145230.3 | V-type proton ATPase subunit e 2 | ND |  |  |  |
| *ATP6V1A* | NM_001690.3 | V-type proton ATPase catalytic subunit A | ND |  |  |  |
| *ATP6V1B1* | NM_001692.3 | V-type proton ATPase subunit B, kidney isoform | ND |  |  |  |
| *ATP6V1B2* | NM_001693.3 | V-type proton ATPase subunit B, brain isoform | c.463+6T>G | splice_region | p.? | rs7460146 |
| *ATP6V1C1* | NM_001695.5 | V-type proton ATPase subunit C 1 | ND |  |  |  |
| *ATP6V1C2* | NM_144583.3 | V-type proton ATPase subunit C 2 | c.1056+15A>C | intronic |  | rs1686482 |
| *ATP6V1D* | NM_015994.3 | V-type proton ATPase subunit D | ND |  |  |  |
| *ATP6V1E1* | NM_001696.4 | V-type proton ATPase subunit E 1 | ND |  |  |  |
| *ATP6V1E2* | NM_001318063.1 | V-type proton ATPase subunit E 2 | ND |  |  |  |
| *ATP6V1F* | NM_004231.4 | V-type proton ATPase subunit F | ND |  |  |  |
| *ATP6V1G1* | NM_004888.4 | V-type proton ATPase subunit G 1 | ND |  |  |  |
| *ATP6V1G2* | NM_138282.2 | V-type proton ATPase subunit G 2 | ND |  |  |  |
| *ATP6V1G3* | NM_133262.3 | V-type proton ATPase subunit G 3 | ND |  |  |  |
| *ATP6V1H* | NM_213619.2 | V-type proton ATPase subunit H | ND |  |  |  |
| *B2M* | NM_004048.4 | Beta-2-microglobulin | ND |  |  |  |
| *BCL2* | NM_000657.2 | Apoptosis regulator Bcl-2 | ND |  |  |  |
| *BDH2* | NM_020139.4 | 3-hydroxybutyrate dehydrogenase type 2 | ND |  |  |  |
| *BLVRA* | NM_000712.3 | Biliverdin reductase A | c.7G>A | missense | p.A3T | rs699512 |
| *BLVRB* | NM_000713.2 | Flavin reductase (NADPH) | ND |  |  |  |
| *BMP2* | NM_001200.3 | Bone morphogenetic protein 2 | ND |  |  |  |
| *BMP4* | NM_001202.5 | Bone morphogenetic protein 4 | ND |  |  |  |
| *BMP6* | NM_001718.5 | Bone morphogenetic protein 6 | ND |  |  |  |
| *BOLA3* | NM_212552.3 | BolA-like protein 3 | ND |  |  |  |
| *BTBD9* | NM_052893.2 | BTB/POZ domain-containing protein 9 | ND |  |  |  |
| *CACNA1C* | NM_001129839.1 | Voltage-dependent L-type calcium channel subunit alpha-1C | c.5505G>A | synonymous | p.= | rs1051375 |
|  |  |  | c.5459C>T | missense | p.P1820L | rs10848683 |
| *CACNA1G* | NM_198387.2 | Voltage-dependent T-type calcium channel subunit alpha-1G | ND |  |  |  |
| *CAND1* | NM_018448.4 | Cullin-associated NEDD8-dissociated protein 1 | ND |  |  |  |
| *CALR* | NM_004343.3 | Calreticulin | ND |  |  |  |
| *CD163* | NM_004244.5 | Scavenger receptor cysteine-rich type 1 protein M130 | c.1024A>G | missense | p.I342V | rs4883263 |
| *CIAO1* | NM_004804.3 | Probable cytosolic iron-sulfur protein assembly protein CIAO1 | ND |  |  |  |
| *CIAO2A* | NM_001014812.2 | Cytosolic iron-sulfur assembly component 2A | c.117A>G | synonymous | p.= | rs332259 |
| *CIAO2B* | NM_016062.4 | Cytosolic iron-sulfur assembly component 2B | ND |  |  |  |
| *CIAO3* | NM_022493.2 | Cytosolic iron-sulfur assembly component 3 | ND |  |  |  |
| *CIAPIN1* | NM_020313.4 | Anamorsin | ND |  |  |  |
| *COX15* | NM_004376.6 | Cytochrome c oxidase assembly protein COX15 homolog | ND |  |  |  |
| *CP* | NM_000096.3 | Ceruloplasmin | c.2662-12T>C | intronic |  | rs16861582 |
| *CPOX* | NM_000097.7 | Oxygen-dependent coproporphyrinogen-III oxidase, mitochondrial | ND |  |  |  |
| *CUBN* | NM_001081.3 | Cubilin | c.8150C>G | missense | p.S2717W | rs2796835 |
|  |  |  | c.6485G>A | missense | p.C2162Y | rs1276712 |
|  |  |  | c.4675C>T | missense | p.P1559S | rs1801231 |
|  |  |  | c.4563T>A | synonymous | p.= | rs1801229 |
|  |  |  | c.2791+9C>T | intronic |  | rs10795440 |
|  |  |  | c.758T>C | missense | p.F253S | rs1801222 |
|  |  |  | c.123-16C>G | intronic |  | rs2281648 |
| *CUL1* | NM_003592.3 | Cullin-1 | ND |  |  |  |
| *CYBRD1* | NM_024843.3 | Cytochrome b reductase 1 | ND |  |  |  |
| *DHCR7* | NM_001163817.1 | 7-dehydrocholesterol reductase | c.1272C>T | synonymous | p.= | rs909217 |
|  |  |  | c.1158T>C | synonymous | p.= | rs760241 |
|  |  |  | c.964-67C>T | intronic |  | rs1792268 |
|  |  |  | c.438T>C | synonymous | p.= | rs949177 |
|  |  |  | c.207T>C | synonymous | p.= | rs1790334 |
|  |  |  | c.189G>A | synonymous | p.= | rs1044482 |
| *ELAC1* | NM_018696.3 | Zinc phosphodiesterase ELAC protein 1 | ND |  |  |  |
| *EPAS1* | NM_001430.4 | Endothelial PAS domain-containing protein 1 | c.218-10_218-8delCCC | splice_region | p.? |  |
| *EPO* | NM_000799.4 | Erythropoietin | ND |  |  |  |
| *EPOR* | NM_000121.4 | Erythropoietin receptor | ND |  |  |  |
| *ERFE* | NM_001291832.2 | Erythroferrone | ND |  |  |  |
| *EXOC6* | NM_001319200.1 | Exocyst complex component 6 | c.1973+17dupA | intronic |  | rs60582805 |
|  |  |  | c.1172C>T | missense | p.T391I | rs1326331 |
| *FBXL5* | NM_012161.4 | F-box/LRR-repeat protein 5 | ND |  |  |  |
| *FDX1* | NM_004109.5 | Adrenodoxin, mitochondrial | ND |  |  |  |
| *FDX2* | NM_001031734.3 | Ferredoxin-2, mitochondrial | c.159T>G | synonymous | p.= | rs378395 |
|  |  |  | c.45A>G | synonymous | p.= | rs395782 |
| *FDXR* | NM_001258015.2 | NADPH:adrenodoxin oxidoreductase, mitochondrial | c.273+201C>T | intronic |  | rs2070918 |
| *FECH* | NM_001012515.2 | Ferrochelatase, mitochondrial | c.939A>G | synonymous | p.= | rs536560 |
|  |  |  | c.798C>G | synonymous | p.= | rs536765 |
|  |  |  | c.332+23A>G | intronic |  | rs577152 |
| *FOLR1* | NM_016725.3 | Folate receptor alpha | ND |  |  |  |
| *FLVCR1* | NM_014053.3 | Feline leukemia virus subgroup C receptor-related protein 1 | ND |  |  |  |
| *FTH1* | NM_002032.3 | Ferritin heavy chain | ND |  |  |  |
| *FTL* | NM_000146.4 | Ferritin light chain | ND |  |  |  |
| *FTMT* | NM_177478.2 | Ferritin, mitochondrial | ND |  |  |  |
| *FXN* | NM_000144.4 | Frataxin, mitochondrial | c.54A>G | synonymous | p.= | rs2481598 |
| *GAST* | NM_000805.4 | Gastrin | ND |  |  |  |
| *GDF15* | NM_004864.3 | Growth/differentiation factor 15 | ND |  |  |  |
| *GFER* | NM_005262.2 | FAD-linked sulfhydryl oxidase ALR | ND |  |  |  |
| *GLRX3* | NM_001199868.2 | Glutaredoxin-3 | ND |  |  |  |
| *GLRX5* | NM_016417.2 | Glutaredoxin-related protein 5, mitochondrial | ND |  |  |  |
| *GSTP1* | NM_000852.3 | Glutathione S-transferase P | ND |  |  |  |
| *HAAO* | NM_012205.2 | 3-hydroxyanthranilate 3,4-dioxygenase | c.109A>G | missense | p.I37V | rs3816183 |
|  |  |  | c.81-10C>T | intronic |  | rs3816184 |
| *HAMP* | NM_021175.4 | Hepcidin | ND |  |  |  |
| *HAO1* | NM_017545.3 | Hydroxyacid oxidase 1 | ND |  |  |  |
| *HEPH* | NM_138737.4 | Hephaestin | c.116T>C | missense | p.V39A | rs5919015 |
|  |  |  | c.634+15A>G | intronic |  | rs5918591 |
|  |  |  | c.2239+15A>G | intronic |  | rs1264215 |
|  |  |  | c.1307G>A | missense | p.S436N | rs143121749 |
| *HEPHL1* | NM_001098672.1 | Hephaestin-like protein 1 |  | upstream_gene |  | rs2511403 |
|  |  |  | c.751A>G | missense | p.N251D | rs1945783 |
|  |  |  | c.1232+9C>G | intronic |  | rs1878799 |
| *HFE* | NM_000410.4 | Hereditary hemochromatosis protein | ND |  |  |  |
| *HIF1A* | NM_181054.2 | Hypoxia-inducible factor 1-alpha | ND |  |  |  |
| *HIF1AN* | NM_017902.3 | Hypoxia-inducible factor 1-alpha inhibitor | ND |  |  |  |
| *HJV* | NM_213653.4 | Hemojuvelin | ND |  |  |  |
| *HMBS* | NM_001258209.1 | Porphobilinogen deaminase | ND |  |  |  |
| *HMOX1* | NM_002133.3 | Hem oxygenase 1 | ND |  |  |  |
| *HMOX2* | NM_001286271.1 | Hem oxygenase 2 | ND |  |  |  |
| *HP* | NM_001126102.2 | Haptoglobin | ND |  |  |  |
| *HPR* | NM_020995.3 | Haptoglobin-related protein | c.1015C>G | missense | p.H339D | rs12646 |
| *HPX* | NM_000613.2 | Hemopexin | ND |  |  |  |
| *HSCB* | NM_172002.5 | Iron-sulfur cluster co-chaperone protein HscB | ND |  |  |  |
| *HSPA9* | NM_004134.6 | Stress-70 protein, mitochondrial | ND |  |  |  |
| *IBA57* | NM_001010867.3 | Putative transferase CAF17, mitochondrial | ND |  |  |  |
| *IDO1* | NM_002164.5 | Indoleamine 2,3-dioxygenase 1 | c.857-28G>A | intronic |  | rs3739319 |
| *IL6* | NM_000600.5 | Interleukin-6 | ND |  |  |  |
| *IREB2* | NM_004136.3 | Iron-responsive element-binding protein 2 | c.475G>C | missense | p.V159L | rs2958720 |
|  |  |  | c.629+22A>C | intronic |  | rs2938672 |
|  |  |  | c.1739T>C | missense | p.I580T | rs2230940 |
|  |  |  | c.2472+12A>C | intronic |  | rs4887060 |
|  |  |  | c.2616C>T | synonymous | p.= | rs13180 |
| *ISCA1* | NM_030940.4 | Iron-sulfur cluster assembly 1 homolog, mitochondrial | ND |  |  |  |
| *ISCA2* | NM_194279.4 | Iron-sulfur cluster assembly 2 homolog, mitochondrial | ND |  |  |  |
| *ISCU* | NM_001301140.1 | Iron-sulfur cluster assembly enzyme ISCU, mitochondrial | c.19T>G | missense | p.F7V | rs10778647 |
|  |  |  | c.20T>G | missense | p.F7C | rs10778648 |
| *ITLN1* | NM_017625.3 | Intelectin-1 | ND |  |  |  |
| *KDM3A* | NM_001146688.1 | Lysine-specific demethylase 3A | ND |  |  |  |
| *LCN2* | NM_005564.4 | Neutrophil gelatinase-associated lipocalin | c.476-28A>G | intronic |  | rs2232629 |
| *LRP1* | NM_002332.2 | Prolow-density lipoprotein receptor-related protein 1 | c.6842-22C>G | intronic |  | rs6581127 |
|  |  |  | c.7278C>T | synonymous | p.= | rs1800139 |
|  |  |  | c.8699A>C | splice_region | p.Q2900P | rs7397167 |
|  |  |  | c.8702-5C>T | splice_region | p.? | rs1800189 |
|  |  |  | c.8893-30T>C | intronic |  | rs7308552 |
|  |  |  | c.8997T>C | synonymous | p.= | rs7308698 |
|  |  |  | c.9783G>A | synonymous | p.= | rs1140648 |
| *LRP2* | NM_004525.2 | Low-density lipoprotein receptor-related protein 2 | c.13113C>T | synonymous | p.= | rs990626 |
|  |  |  | c.79+15A>G | intronic |  | rs1559013 |
|  |  |  | c.63G>C | synonymous | p.= | rs1559014 |
| *LRP3* | NM_002333.3 | Low-density lipoprotein receptor-related protein 3 | c.1032T>C | synonymous | p.= | rs2112800 |
|  |  |  | c.2123T>C | missense | p.V708A | rs3745974 |
| *LTF* | NM_001199149.1 | Lactotransferrin | ND |  |  |  |
| *LYRM4* | NM_001164840.2 | LYR motif-containing protein 4 | ND |  |  |  |
| *MCOLN1* | NM_020533.2 | Mucolipin-1 | ND |  |  |  |
| *MELTF* | NM_005929.5 | Melanotransferrin | c.644+30A>C | intronic |  | rs2288766 |
| *MMS19* | NM_001330128.1 | MMS19 nucleotide excision repair protein homolog | c.2280C>T | synonymous | p.= | rs2152092 |
|  |  |  | c.1130-28T>G | intronic |  | rs29001315 |
|  |  |  | c.847-9C>T | intronic |  | rs11592973 |
|  |  |  | c.262+651G>A | intronic |  | rs7897727 |
|  |  |  | c.203C>G | missense | p.A68G | rs2275586 |
| *MON1A* | NM_032355.3 | Vacuolar fusion protein MON1 homolog A | ND |  |  |  |
| *MT-ATP6* | ENST00000361899 | ATP synthase subunit a | m.8702G>A | missense | p.A59T |  |
| *MTF1* | NM_005955.2 | Metal regulatory transcription factor 1 | ND |  |  |  |
| *MYC* | NM_002467.6 | Myc proto-oncogene protein | ND |  |  |  |
| *NCOA4* | NM_001145261.1 | Nuclear receptor coactivator 4 | ND |  |  |  |
| *NDOR1* | NM_001144027.2 | NADPH-dependent diflavin oxidoreductase 1 | c.-6A>G | kozak_sequence |  | rs7032361 |
| *NDUFB11* | NM_019056.7 | NADH dehydrogenase [ubiquinone] 1 beta subcomplex subunit 11, mitochondrial | ND |  |  |  |
| *NDUFV1* | NM_001166102.1 | NADH dehydrogenase [ubiquinone] flavoprotein 1, mitochondrial | ND |  |  |  |
| *NEDD8* | NM_006156.3 | NEDD8 | ND |  |  |  |
| *NFE2L1* | NM_001330262.1 | Endoplasmic reticulum membrane sensor NFE2L1 | ND |  |  |  |
| *NFS1* | NM_021100.5 | Cysteine desulfurase, mitochondrial | ND |  |  |  |
| *NFU1* | NM_015700.3 | NFU1 iron-sulfur cluster scaffold homolog, mitochondrial | ND |  |  |  |
| *NUBP1* | NM_001278506.1 | Cytosolic Fe-S cluster assembly factor NUBP1 | ND |  |  |  |
| *NUBP2* | NM_012225.3 | Cytosolic Fe-S cluster assembly factor NUBP2 | c.136-49G>C | intronic |  | rs2575351 |
|  |  |  | c.174C>A | synonymous | p.= | rs2235648 |
|  |  |  | c.561T>C | synonymous | p.= | rs344359 |
|  |  |  | c.*121C>G | 3_prime_UTR |  | rs1065656 |
|  |  |  | c.*308G>A | 3_prime_UTR |  | rs1065663 |
| *NUBPL* | NM_025152.2 | Iron-sulfur protein NUBPL | ND |  |  |  |
| *PCBP1* | NM_006196.4 | Poly(rC)-binding protein 1 | ND |  |  |  |
| *PCBP2* | NM_005016.6 | Poly(rC)-binding protein 2 | ND |  |  |  |
| *PCBP3* | NM_020528.3 | Poly(rC)-binding protein 3 | ND |  |  |  |
| *PCBP4* | NM_020418.4 | Poly(rC)-binding protein 4 | ND |  |  |  |
| *PGRMC1* | NM_006667.5 | Membrane-associated progesterone receptor component 1 | ND |  |  |  |
| *PGRMC2* | NM_006320.6 | Membrane-associated progesterone receptor component 2 | ND |  |  |  |
| *PLEKHB2* | NM_001100623.2 | Pleckstrin homology domain-containing family B member 2 | ND |  |  |  |
| *PPOX* | NM_000309.4 | Protoporphyrinogen oxidase | ND |  |  |  |
| *PRNP* | NM_001080121.2 | Major prion protein | ND |  |  |  |
| *PUS1* | NM_025215.6 | tRNA pseudouridine synthase A | ND |  |  |  |
| *RFC1* | NM_002913.4 | Replication factor C subunit 1 | c.1998+25C>T | intronic |  | rs2066789 |
|  |  |  | c.4-26G>A | intronic |  | rs4975007 |
| *RHOA* | NM_001664.3 | Transforming protein RhoA | ND |  |  |  |
| *RPS27A* | NM_002954.6 | Ubiquitin-40S ribosomal protein S27a | ND |  |  |  |
| *SCARA5* | NM_173833.5 | Scavenger receptor class A member 5 | ND |  |  |  |
| *SDHB* | NM_003000.2 | Succinate dehydrogenase [ubiquinone] iron-sulfur subunit, mitochondrial | c.201-36G>T | intronic |  | rs1022580 |
|  |  |  | c.73-302G>A | intronic |  | rs732679 |
|  |  |  | c.18C>A | synonymous | p.= | rs2746462 |
| *SFXN1* | NM_022754.7 | Sideroflexin-1 | ND |  |  |  |
| *SFXN2* | NM_178858.5 | Sideroflexin-2 | c.-10300C>T | 5_prime_UTR_intronic |  | rs2248418 |
| *SFXN3* | NM_030971.4 | Sideroflexin-3 | ND |  |  |  |
| *SFXN4* | NM_213649.1 | Sideroflexin-4 | c.472-24T>A | intronic |  | rs2420494 |
|  |  |  | c.354A>G | synonymous | p.= | rs2181118 |
| *SFXN5* | NM_001330400.2 | Sideroflexin-5 | ND |  |  |  |
| *SKP1* | NM_170679.3 | SKP1-like protein 1A | ND |  |  |  |
| *SLC11A1* | NM_000578.3 | Natural resistance-associated macrophage protein 1 | c.825A>G | synonymous | p.= | rs2695342 |
|  |  |  | c.1165-16T>C | intronic |  | rs2045433 |
| *SLC11A2* | NM_001174125.2 | Natural resistance-associated macrophage protein 2 | c.1576-14A>G | intronic |  | rs161044 |
|  |  |  | c.184-778T>C | intronic |  | rs224590 |
|  |  |  | c.17A>C | missense | p.Y6S | rs445520 |
|  |  |  | c.34+11G>A | intronic |  | rs427020 |
| *SLC17A1* | NM_005074.3 | Sodium-dependent phosphate transport protein 1 | ND |  |  |  |
| *SLC19A2* | NM_006996.3 | Thiamine transporter 1 | ND |  |  |  |
| *SLC22A17* | NM_020372.3 | Solute carrier family 22 member 17 | ND |  |  |  |
| *SLC25A28* | NM_031212.3 | Mitoferrin-2 | ND |  |  |  |
| *SLC25A37* | NM_016612.3 | Mitotransferrin-1 | ND |  |  |  |
| *SLC25A38* | NM_017875.4 | Mitochondrial glycine transporter | ND |  |  |  |
| *SLC39A8* | NM_001135147.1 | Zinc transporter ZIP8 | ND |  |  |  |
| *SLC39A14* | NM_001128431.4 | Zinc transporter ZIP14 | ND |  |  |  |
| *SLC40A1* | NM_014585.5 | Solute carrier family 40 member 1 (Ferroportin-1) | ND |  |  |  |
| *SLC46A1* | NM_080669.6 | Proton-coupled folate transporter | c.1142+1delT | essential_splice_site | p.? | rs5819844 |
| *SMAD1* | NM_005900.3 | Mothers against decapentaplegic homolog 1 | ND |  |  |  |
| *SMAD4* | NM_005359.5 | Mothers against decapentaplegic homolog 4 | ND |  |  |  |
| *SMAD5* | NM_001001419.2 | Mothers against decapentaplegic homolog 5 | c.1313-1_1313insC | essential_splice_site | p.? | rs55765823 |
| *SOD1* | NM_000454.5 | Superoxide dismutase [Cu-Zn] | ND |  |  |  |
| *SOD2* | NM_001322817.1 | Superoxide dismutase [Mn], mitochondrial | ND |  |  |  |
| *SRI* | NM_003130.4 | Sorcin | ND |  |  |  |
| *STEAP1* | NM_012449.2 | Metalloreductase STEAP1 | ND |  |  |  |
| *STEAP2* | NM_001040665.1 | Metalloreductase STEAP2 | c.1425G>T | missense | p.M475I | rs194525 |
|  |  |  | c.1185+2592C>T | intronic |  | rs194526 |
| *STEAP3* | NM_138637.2 | Metalloreductase STEAP3 | ND |  |  |  |
| *TCIRG1* | NM_006019.3 | V-type proton ATPase 116 kDa subunit a isoform 3 | c.597G>C | synonymous | p.= | rs2471829 |
| *TF* | NM_001063.3 | Serotransferrin | c.1342A>G | missense | p.I448V | rs2692696 |
| *TFR2* | NM_003227.3 | Transferrin receptor protein 2 | ND |  |  |  |
| *TFRC* | NM_001313965.1 | Transferrin receptor protein 1 | c.1468+25G>T | intronic |  | rs507131 |
|  |  |  | c.444+9A>G | intronic |  | rs480760 |
| *TMPRSS6* | NM_001289001.1 | Transmembrane protease serine 6 | c.2180T>C | missense | p.V727A | rs855791 |
|  |  |  | c.1536C>T | synonymous | p.= | rs4820268 |
|  |  |  | c.836+23A>G | intronic |  | rs2235326 |
| *TNF* | NM_000594.3 | Tumor necrosis factor | ND |  |  |  |
| *TRNT1* | NM_182916.2 | CCA tRNA nucleotidyltransferase 1, mitochondrial | c.68C>T | missense | p.P23L | rs334773 |
|  |  |  | c.888A>G | synonymous | p.= | rs1705805 |
| *TRPC6* | NM_004621.5 | Short transient receptor potential channel 6 | ND |  |  |  |
| *TSPO* | NM_000714.5 | Translocator protein | c.439A>G | missense | p.T147A | rs6971 |
|  |  |  | c.485G>A | missense | p.R162H | rs6972 |
| *TST* | NM_003312.5 | Rhodanese | ND |  |  |  |
| *TTYH1* | NM_001201461.1 | Protein tweety homolog 1 | c.735-14C>T | intronic |  | rs889149 |
|  |  |  | c.1188A>G | synonymous | p.= | rs3745420 |
| *TWSG1* | NM_020648.5 | Twisted gastrulation protein homolog 1 | ND |  |  |  |
| *UBA52* | NM_001033930.2 | Ubiquitin-60S ribosomal protein L40 | ND |  |  |  |
| *UBB* | NM_018955.3 | Polyubiquitin-B | ND |  |  |  |
| *UBC* | NM_021009.6 | SUMO-conjugating enzyme UBC9 | c.568T>C | missense | p.S190P | rs7137123 |
| *UGT1A4* | NM_007120.2 | UDP-glucuronosyltransferase 1-4 | c.471T>C | synonymous | p.= | rs2011404 |
| *UROD* | NM_000374.4 | Uroporphyrinogen decarboxylase | ND |  |  |  |
| *UROS* | NM_000375.2 | Uroporphyrinogen-III synthase | ND |  |  |  |
| *YARS2* | NM_001040436.2 | Tyrosine--tRNA ligase, mitochondrial | ND |  |  |  |

ND – not detected
